# Spatial clustering and risk factors of malaria infections in Bata district, Equatorial Guinea

**DOI:** 10.1186/s12936-017-1794-z

**Published:** 2017-04-12

**Authors:** Diana Gómez-Barroso, Emely García-Carrasco, Zaida Herrador, Policarpo Ncogo, María Romay-Barja, Martín Eka Ondo Mangue, Gloria Nseng, Matilde Riloha, Maria Angeles Santana, Basilio Valladares, Pilar Aparicio, Agustín Benito

**Affiliations:** 1 CIBERESP, National Centre of Epidemiology, Carlos III Institute of Health (ISCIII), Madrid, Spain; 2 RICET, National Center of Tropical Medicine, Carlos III Institute of Health (ISCIII), Madrid, Spain; 3 Reference Centre for Endemic Control of Equatorial Guinea (CRCE), Malabo, Equatorial Guinea; 4 Ministry of Health and Social Welfare, Malabo, Equatorial Guinea; 5 University Institute for Tropical Diseases and Public Health of Canarias, Tenerife, Spain

**Keywords:** Malaria, Spatial, Equatorial Guinea, Children, Rapid diagnostic tests

## Abstract

**Background:**

The transmission of malaria is intense in the majority of the countries of sub-Saharan Africa, particularly in those that are located along the Equatorial strip. The present study aimed to describe the current distribution of malaria prevalence among children and its environment-related factors as well as to detect malaria spatial clusters in the district of Bata, in Equatorial Guinea.

**Methods:**

From June to August 2013 a representative cross-sectional survey using a multistage, stratified, cluster-selected sample was carried out of children in urban and rural areas of Bata District. All children were tested for malaria using rapid diagnostic tests (RDTs). Results were linked to each household by global position system data. Two cluster analysis methods were used: hot spot analysis using the Getis-Ord Gi statistic, and the SaTScan™ spatial statistic estimates, based on the assumption of a Poisson distribution to detect spatial clusters. In addition, univariate associations and Poisson regression model were used to explore the association between malaria prevalence at household level with different environmental factors.

**Results:**

A total of 1416 children aged 2 months to 15 years living in 417 households were included in this study. Malaria prevalence by RDTs was 47.53%, being highest in the age group 6–15 years (63.24%, p < 0.001). Those children living in rural areas were there malaria risk is greater (65.81%) (p < 0.001). Malaria prevalence was higher in those houses located <1 km from a river and <3 km to a forest (IRR: 1.31; 95% CI 1.13–1.51 and IRR: 1.44; 95% CI 1.25–1.66, respectively). Poisson regression analysis also showed a decrease in malaria prevalence with altitude (IRR: 0.73; 95% CI 0.62–0.86). A significant cluster inland of the district, in rural areas has been found.

**Conclusions:**

This study reveals a high prevalence of RDT-based malaria among children in Bata district. Those households situated in inland rural areas, near to a river, a green area and/or at low altitude were a risk factor for malaria. Spatial tools can help policy makers to promote new recommendations for malaria control.

## Background

Nearly half of the world’s population live in areas in which they are at risk of one or more vector-borne diseases; approximately 3.3 billion people worldwide are at risk of malaria alone. With 212 million malaria cases in 2015 and 429,000 deaths, malaria is the most common vector-borne infectious disease, with sub-Saharan Africa carrying most of the burden [1]. The causal protozoon *Plasmodium* is transmitted from person to person through the bite of adult female *Anopheles* mosquitoes. In regions of stable transmission, children are at highest risk of becoming symptomatic after infection with malaria parasites [1, 2].

The transmission of malaria is intense in the majority of the countries of sub-Saharan Africa, particularly in those that are located along the Equatorial strip [3]. Studies mapping potential mosquito habitats, transmission risk, or disease prevalence have been performed in Africa where malaria transmission not only varies from one country to another, but also local differences in time and space exist [3–5]. Moreover, internal geographical differences are common, together with transmission differences associated with urban development. Actually, some studies have concluded that the burden of malaria is significantly lower in large cities than in rural areas of sub-Saharan Africa [6, 7].

The population of Equatorial Guinea (EG) is exposed to one of the highest levels of malaria infection in the world, especially in the mainland [8, 9]. Malaria transmission is stable throughout the year in EG [10]. The tropical, year-round, humid climate and the many rivers and streams, both fast and slow flowing, provide ideal breeding conditions for different malaria vectors [11]. The current malaria burden differs between the mainland and the island, being considerably higher in the continent, where less control activities have been implemented [12]. Furthermore, no control initiatives exist in the continental area since the EG Malaria Control Initiative (EGMCI) was suspended in 2011 due to funding restrictions [8] Reliable information about malaria transmission risk is essential for understanding variations in local disease epidemiology and to stratify intervention programs. Thus, the present study aimed at describing the current prevalence of malaria among children, to study their environmental related factors and to detect spatial clusters in Bata district, in EG.

## Methods

### Study area and population

**Fig. 1:**
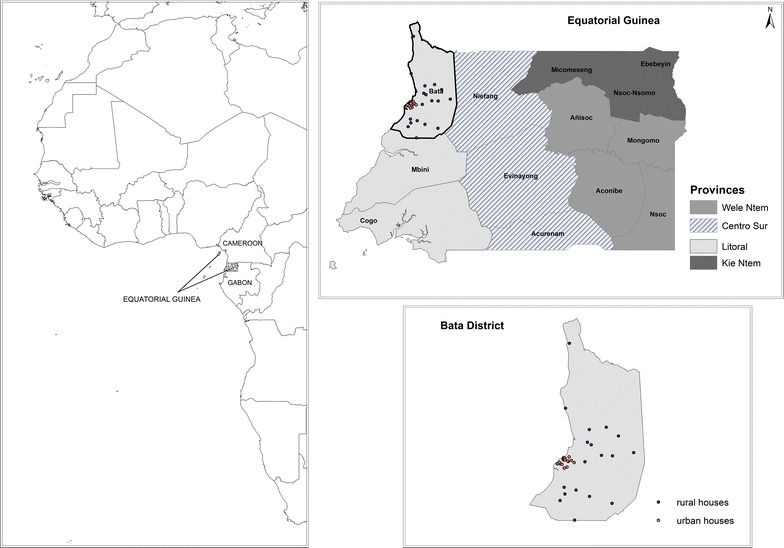
Equatorial Guinea administrative division and Spatial distribution visited households in Bata district, Equatorial Guinea

The EG mainland continental region covers 26,017 sq km and is bordered by Cameroon in the north and Gabon in the south and east. EG consists of two parts, an insular and a mainland region. Bioko Island is the northernmost part of EG and is the site of the country’s capital, Malabo. The continental region has a population of 882,747, mainly ethnic Fang tribes [13]. It is composed of four provinces: Centro Sur, Kie-Ntem, Litoral, and Wele-Nzas. Each province is divided into several districts (Fig. 1). Bata district, situated in Litoral Province, is the largest district in the continental area (230,283 inhabitants) and has a tropical climate with two dry seasons (December to March and June to September) alternating with two rainy seasons (March to June and September to December). Mean daily maximum and minimum temperatures range between 29 and 32 °C and 19–22 °C, respectively.

The current study is part of a survey conducted by the Health Institute Carlos III and the Equatoguinean Ministry of Health and Social Welfare (MINSABS in Spanish) as part of the project called ‘Prevamal’. This project aimed at providing baseline data on malaria prevalence and practices and attitudes among the targeted population. Other methodological aspects have been described elsewhere [14].

Sampling was carried out with the use of a multistage, stratified cluster strategy. The strata were rural and urban settings and three age groups (2–12 months old, 13 months–5 years old and 6–15 years old), using an expected malaria prevalence of 50%. The initial sample size was increased in prevision of missing data but replacement was not carried out at any of the sampling stages [15].

Firstly, rural villages (n = 21) and urban neighbourhoods (n = 23) were randomly selected with probability weights proportional to population size. Secondly, sampling units were randomly selected households from an updated census from each cluster, which was previously provided by the head of the village or neighbourhood. All children with reported age between 2 months and 15 years living in the selected household and not receiving malaria treatment at the moment of the survey were included in the study.

### Data collection

Between mid-June and mid-August 2013, four teams of three trained medical personnel visited each village/neighbourhood for 2 consecutive days to collect data. A closed-end, pre-piloted questionnaire was administered to the household head/caretaker of participating children. The questionnaire comprised the following: demographic characteristics, health status, and history of previous episodes of malaria and malaria-related behaviours. The questionnaire was translated into the main local language, Fang, and the option was given to the care provider to be interviewed in Fang or in Spanish, which is one of the official languages in the country. Each individual received a unique code number.

NADAL^®^ rapid diagnostic test (Nal von Minden, Moers, Germany) for malaria infection was performed in situ. Malaria positive cases were treated according to the national treatment guidelines. Children’s household geographic coordinates (latitude and longitude) were marked using a hand-held GPS (Garmin Dakota 20^®^), and processed with Google Earth.

The data source was used to analyse the environmental conditions in the study area, mostly obtained in shape file format from the interactive Atlas of the Republic of Equatorial Guinea [16]: the hydrographic network, information on protected areas, areas of community forests, national forests and forest areas, and the digital terrain model (DTM). Information on protected areas, forests and forest areas were aggregated in a single variable called green zone.

The collected data were double entered into a data entry file using EpiData software, V.3.1. All records were given a unique identification code. Epidemiologic information and laboratory results were linked to each household’s GPS data.

### Statistical analysis

Frequencies and percentages were used to summarize data and to explore the differences by children’s sex, age, setting and ethnic group.

Siblings from the same family shared the same location. Data were aggregated by household to describe the characteristics of the distribution of malaria prevalence among the study subjects and selected households. For each corrected location, data were subsequently aggregated, and the number of recorded malaria episodes and number of susceptible were calculated. After aggregating data at the house block level, the percentage of malaria infections per household was computed.

To assess environmental risk factors, 1-km buffer around each river and 3-km buffer around every defined green area were created to assess the distance to both from the sampled households. The elevation data were obtained from the DTM, defining 100 m as the cut-off value to assess environmental risk.

In order to study the associations between malaria prevalence at household level and potential explanatory variables, Poisson regression models were used. First univariable models for each potential predictor were fitted. Second a multivariable model was constructed using a backward stepwise procedure. Variables with a p value less than 0.20 in the univariable analyses was selected to entry into the multivariable model.

Regression was performed using Stata version 11.0 (College Station, TX, USA).

### Clusters analysis

With the aim of checking clusters in the space of houses with higher prevalence rates two different cluster analysis methods were used. Firstly, a hot spot analysis using the Getis-Ord Gi statistic has been carried out. This tool works by looking at each feature within the context of neighbouring features. A feature with a high value is interesting but may not be a statistically significant hot spot. To be a statistically significant hot spot, a feature will have a high value and be surrounded by other features with high values as well. The local sum for a feature and its neighbours is compared proportionally to the sum of all features; when the local sum is very different from the expected local sum, and that difference is too large to be the result of random chance, statistically significant z-score results. The resultant z-scores and p values tell where features with either high or low values cluster spatially [17]. The method, SaTScan™ purely spatial statistic estimator developed by Kulldorff [18], based on the assumption of a Poisson distribution to detect spatial clusters, has been used. This method consists of creating a circular window, which scans the entire study area. In this study, the spatial window has been restricted to a maximum radius of the average distance between cases. The radius of the centroid varies continuously in size from 0 to the specified upper limit. An infinite number of overlapping windows of different sizes and shapes is thus generated, which together covers the entire study area. Each circle is a possible candidate cluster. The null hypothesis that risk is constant in space is tested against the alternative hypothesis: the risk is different in at least one circle (or window). For each circle, the number of observed cases inside and outside the window is counted along with the number of expected cases, estimated according to the model. On this basis, the likelihood within each circle is then calculated. The circle having the maximum likelihood and containing more cases than expected is denominated the most likely cluster.

$$\left( {\frac{c}{E[c]}} \right)^{c} \left( {\frac{{C - c}}{{C- E[c]}}} \right)^{{C-c}} I()$$

where C is the total number of cases, c is the observed number of cases within the window, and E[c] is the covariate-adjusted expected number of cases within the window under the null-hypothesis. I() is an indicator function, which is equal to 1 in cases where the window has more cases than those expected under the null hypothesis, and 0 otherwise.

The increase in observed cases above the number expected was assessed using Monte Carlo test simulations (999 replications) with a 95% confidence interval.

ArcGIS 10.1 and SaTScan 9 were used to do the spatial analysis and to produce the maps.

### Ethical clearance

The study was approved by the ethical review board of the Health Institute Carlos III (ISCIII in Spanish) and the Minister of Health and Social Welfare of Equatorial Guinea (MINSABS). Support letters were obtained from MINSABS and the Hospital of Bata. The village and neighbourhoods representatives were informed by an official letter from MINSABS of the day of the visit and the scope of the study. Written informed consent was obtained from all patients prior to study inclusion. Anonymity was assured. A written statement was also included on the introductory part of the questionnaires in which further information concerning the purpose of the study and the confidentiality of the research information was given. Data were analysed in anonymous form.

## Results

Overall, 444 households were surveyed, out of which 27 were excluded for not having the exact coordinates for its spatial location. All children aged 2 months–15 years old living in the randomly selected households were included in the study. Afterwards, 13 children were excluded from the analysis for not having the rapid test result recorded in the database. A total of 1416 children from 417 households were analysed (mean number of population per household: three ranged from 1 to 15) out of which 50.64% were females and 49.36% males. The median age was 4 years old. The spatial distribution of the 417 visited households is shown in Fig. 1.

The malaria prevalence by rapid diagnostic test (RDTs) was 47.53% (95% CI 45.0–50.2). The overall prevalence varied between villages and neighbourhoods, especially in rural sites (ranges varied from 30.77 to 89.74% and from 20 to 48.57% in rural and urban areas, respectively).

Malaria prevalence was similar in girls (46.30%) than in boys (48.78%). The highest prevalence was found in the age group 6–15 years old (63.24%) while children aged 2–12 months were less affected (25.44%; p < 0.001). Those children living in rural areas were more at risk of malaria at the time of the survey than children living in urban neighbourhoods (65.81 vs 36.12%, respectively, p < 0.001). The study children belonging to the Fang ethnic group were more frequently infected by malaria than those from the Combe ethnic group (49.41 and 31.73%, respectively, p = 0.001).

### Factors related to malaria prevalence at household level

The results of the multivariate analysis showed that households located in rural areas were more likely to report higher malaria prevalence than those located in urban zones (IRR = 2.18; 95% CI 1.86–2.57). If a house belonged to a Combe ethnic group, children were less likely to have malaria than those belonging to the predominant Fang ethnic group (IRR: 0.67; 95% CI 0.52–0.87). Significant associations were also observed between malaria prevalence and proximity to a river and a forest: malaria prevalence at household level was higher in those houses located <1 km from a river and 3 km from the forest (IRR: 1.31; 95% CI 1.13–1.51 and IRR: 1.44; 95% CI 1.25–1.66, respectively). Poisson regression analysis also showed a decrease in malaria prevalence with altitude (IRR: 0.73; 95% CI 0.62–0.86) (Table 1).

**Table 1:** Factors associated with malaria prevalence at household level, June–August 2013, Bata district, Equatorial Guinea

Variable	Univariable analysis			Multivariable analysis		
	IRR	(95% CI)	p value	IRR	(95% CI)	p value
River 1 km	1.51	(1.29–0.77)	<0.001	1.31	(1.13–1.51)	<0.001
Forest 1 km	1.78	(1.53–2.06)	<0.001	1.44	(1.25–1.66)	<0.001
Altitude	0.63	(0.55–0.72)	<0.001	0.73	(0.62–0.86)	<0.001
Zone	2.49	(2.11–2.92)	<0.001	2.18	(1.86–2.57)	<0.001
Ethnia combe	0.69	(0.47–0.99)	0.044	0.67	(0.52–0.87)	0.003
Ethnia others	0.87	(0.66–1.15)	0.341	0.93	(0.75–1.17)	0.540

### Cluster analysis

**Fig. 2:**
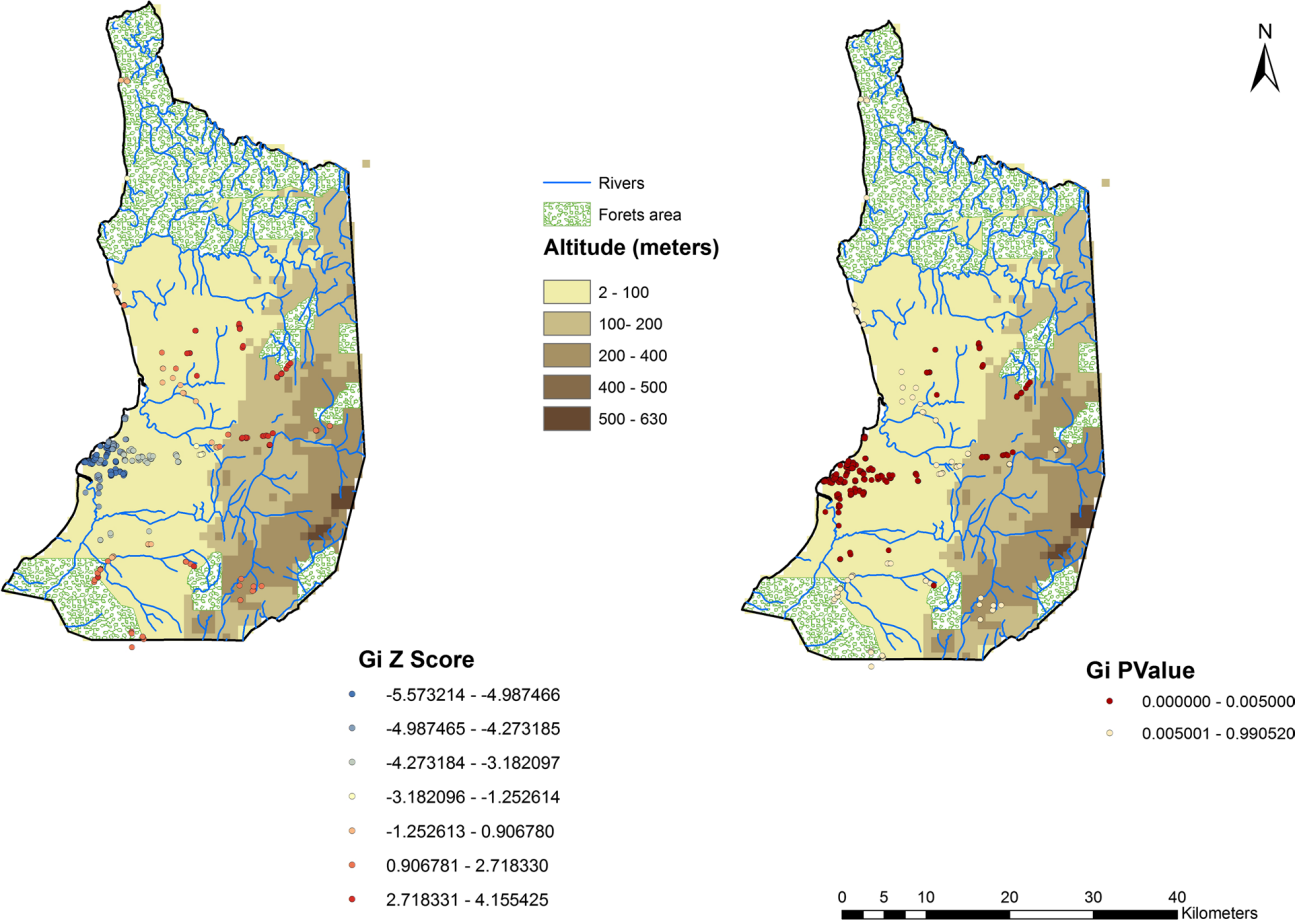
Spatial cluster distribution using the Getis-Ord Gi and Gi p values in Bata district, Equatorial Guinea

The Getis-Ord Gi statistic returned for each feature in the dataset is a z-score. For statistically significant positive z-scores, the larger the z-score is, the more intense the clustering of high values (hot spot). For statistically significant negative z-scores, the smaller the z-score is, the more intense the clustering of low values (cold spot). Figure 2 shows the spatial cluster distribution using the Getis-Ord Gi. The higher z score values, in red, correspond to the houses aggregated with higher prevalence rates. These clusters, mainly located at rural villages, were placed in the inland and at higher elevations. The clusters with the lowest z score values (in blue), representing aggregations of houses with low rates prevalence, were detected close to the sea level. The map with p values shows the statistical significance of the clusters.

**Fig. 3:**
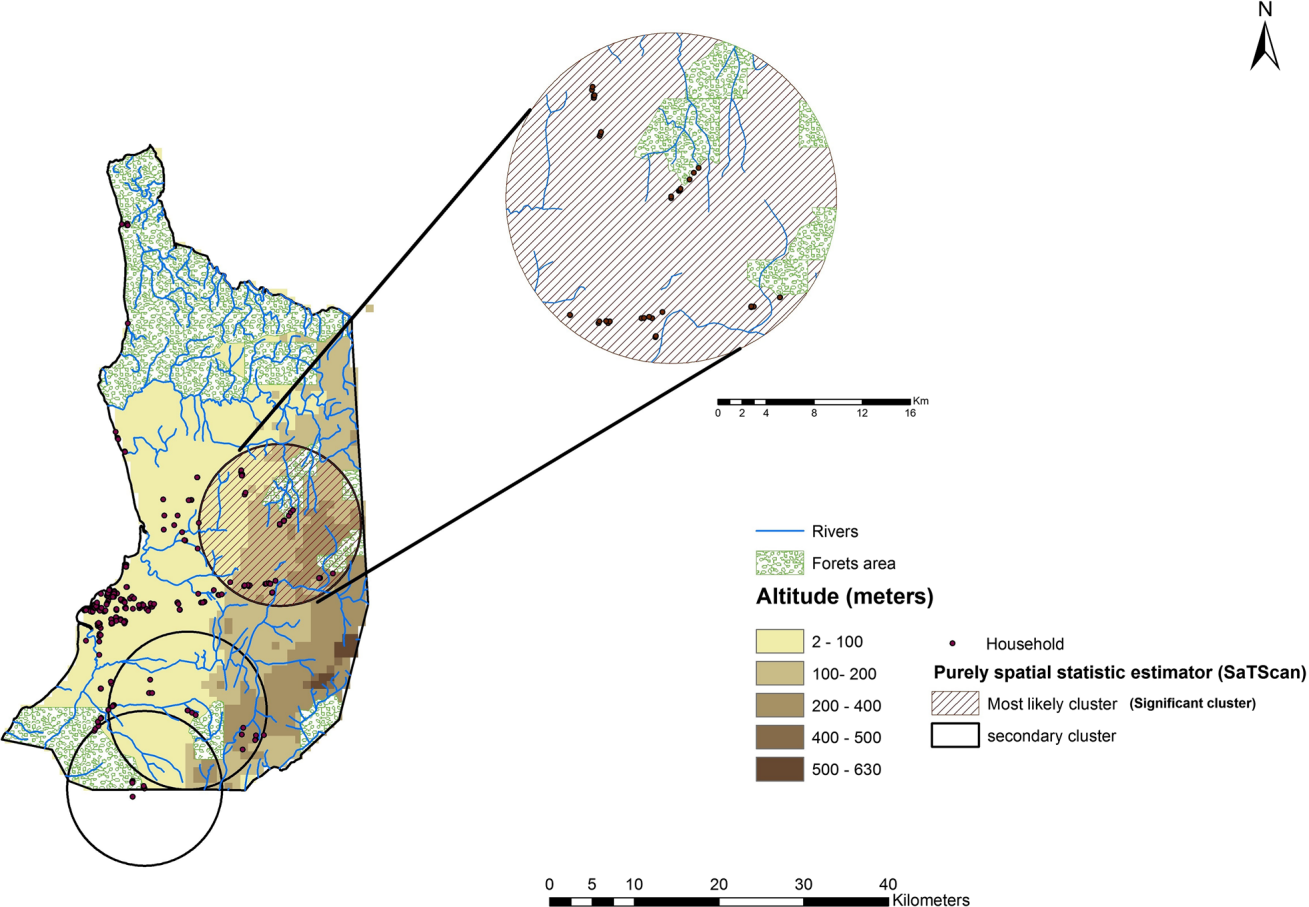
Spatial cluster distribution using Scan statistic in Bata district, Equatorial Guinea

Different distances to detect clusters using Scan statistic (1, 5 and 10 km) have been used. For 1 and 5 km, the clusters detected were not significant, but when it used the spatial window with 10 km, one significant cluster in the inland of the district (Fig. 3) was detected. This cluster included 42 houses and 110 positive cases. The expected cases were 71. The relative risk was 1.65 and the likelihood ratio 10.31 (p value <0.005). In addition, two not significant clusters were detected in the south of the district.

## Discussion

Of the numerous studies investigating risk factors for malaria in sub-Saharan Africa, few have simultaneously examined individual, household and environmental risk factors. This is the first to do so in EG, where prevalence of malaria was 46.2% [15] the underlying individual, household and environmental factors that characterize both malaria risk per se and high-risk areas was investigated. Using spatial statistics and geographical information system (GIS), the spatial distribution of malaria cases among schoolchildren from 21 rural villages and 23 urban neighbourhoods was investigated. High prevalence of malaria, especially in those children living in rural zones has been found.

Significant spatial clusters where the risk of malaria was higher have been identified using both methods. Areas characterized by low altitude and major proximity to rivers and forests were strongly associated with risk of malaria. Several studies have linked the prevalence of malaria with similar environmental factors in other sub-Saharan countries [19–26].

The prevalence of malaria in school-aged children determined by RDT was high (47.5%) in Bata district. This is consistent with results from the WHO Malaria Report 2015 [1]. Children aged under 1 year old were less affected. According to previous research, risk of infection is lower in this age group due to the passive immunity provided by the mother during pregnancy and breast feeding [27]. Moreover, the use of bed nets and other preventative activities are known to be more frequently applied for children under 1 year old in EG [28]. Malaria prevalence progressively increased with age in this study area. This result is consistent with previous research carried out in other African countries with similar prevalence [29]. Fang children had the highest prevalence compared to other ethnic groups. Immuno-epidemiological studies suggest that some African ethnicities are more susceptible to malaria [30–32].

Regarding household risk factors, it was found that those houses at low altitude were at higher risk of malaria. It was known that one of the most important factors influenced by altitude is temperature, which affects both the development and survival of the vector and the development of *Plasmodium* within the vector [20]. Another factor which may be associated with altitude is suitability for mosquito breeding [33]. Living further away from a river and the forest were protective factors against malaria The relationship between malaria incidence and distance to a river and/or a forest (as potential breeding sites) has been observed before [34, 35]. Bata district is a tropical zone rich in vegetation, which provides few breeding sites associated with irrigated areas on the banks of a river. Although this study was carried out during the dry season a high prevalence of malaria and a strong association between malaria and several environmental factors was found. These data support that, despite of the seasonality, malaria follows a year-round transmission on EG mainland.

The most likely cluster detected including 42 houses with 110 positive cases. This area of Bata district has an extensive hydrographic network, forest areas and is between 0 and 200 m altitude. Additionally, two secondary clusters without statistical significance were detected in the south of the district. However, any significant cluster by using 1- and 5-km windows in the scan method was not found. This might be due housing distribution in the area; households in urban areas were gathered together while houses in the rural areas tended to be distanced from each other.

Similar results were obtained by the Getis-Ord Gi statistic method, showing that those areas with higher prevalence were placed in the inland of the district. Cluster analysis has been used in other studies to detect malaria clusters in Africa, allowing detection of risk areas in different parts of the continent [2, 20, 36, 37].

This is the first study carried out in EG so far assessing malaria spatial human prevalence taking into account environmental factors. Previous research was carried out by Cano et al. [38], to assess the spatial distribution of different species of *Anopheles* and its association with related factors in a small village in the mainland region of EG. This study was undertaken in the district of Bata. In this district, there were improvements in malaria control from 2007 to 2012 thanks to the EGMCI. This initiative followed the success of the Bioko Island Malaria Control Project in the insular area of EG [39]. Vector control formed the basis of this initiative and consisted of indoor residual spraying [12]. Unfortunately, the EGMCI was suspended in 2011 due to funding restrictions [8]. While the EGMCI is meeting most of the WHO global goals and targets in the insular area with success, the EGMCI activities were abruptly interrupted. Since then, no major efforts have been taken in malaria control in the continental area. Vector control activities together with better access to malaria first-line treatment will likely be necessary to move forward in malaria prevention and control in this zone. Vector control activities together with better access to malaria first-line treatment will likely be necessary to move forward in malaria prevention and control in this zone.

### Limitations

The present study was conducted in Bata district, and the findings may not be generalizable to the whole country. Additionally, the cross-sectional nature of the data does not allow examining causality in the relationship between malaria prevalence and several risk factors. Climate variables, such as humidity and temperatures not were included in the analysis due to nature of the study. It was conducted during the dry season. Finally, entomological data would have also been desirable to better explain the relationship of malaria prevalence with environmental factors. RDTs were used to estimate malaria prevalence in the study. However, it should be taken into account that discrepancies in prevalence estimates generated from microscopy testing and from RDT testing exist, and that there are difficult to reconcile [12].

## Conclusions

The findings reveal a high prevalence of RDT-based malaria prevalence among children in Bata district. Environmental factors related to high prevalence of disease were found. Malaria continues to be a significant public health problem in the mainland. To effectively tackle malaria, the National Programme to Fight Malaria in Equatorial Guinea should orient interventions to local needs, taking into consideration these geographical risk factors, especially in rural places.

These results will assist national and regional stakeholders in planning and undertaking contextual and evidence-based policy initiatives. The spatial methods allow to know the areas with highest risks and can help policy makers to plan and to undertake new regional initiatives to streamline recommendations. Methods to detect clusters are particularly important to design preventive interventions.

## Abbreviations

RDT: rapid diagnostic test
GPS: global position system
IRR: incidence rate ratio
CI: confidence interval
EG: Equatorial Guinea
EGMCI: Equatorial Guinea Malaria Control Initiative
MINSABS: Equatoguinean Ministry of Health and Social Welfare (in Spanish)
DTM: digital terrain model
AIC: akaike information criterion
ITN: insecticide-treated nets
